# Mass Spectrometric Identification of Proteins Enhanced by the Atomic Force Microscopy Immobilization Surface

**DOI:** 10.3390/ijms22010431

**Published:** 2021-01-04

**Authors:** Anna L. Kaysheva, Pavel A. Frantsuzov, Arthur T. Kopylov, Tatyana O. Pleshakova, Alexander A. Stepanov, Kristina A. Malsagova, Alexander I. Archakov, Yurii D. Ivanov

**Affiliations:** Institute of Biomedical Chemistry, 119121 Moscow, Russia; inst@ibmc.msk.ru (P.A.F.); a.t.kopylov@gmail.com (A.T.K.); t.pleshakova1@gmail.com (T.O.P.); aleks.a.stepanov@gmail.com (A.A.S.); kristina.malsagova86@gmail.com (K.A.M.); alexander.archakov@ibmc.msk.ru (A.I.A.); yurii.ivanov.nata@gmail.com (Y.D.I.)

**Keywords:** smooth chip, mass spectrometry, atomic force microscope, protein detection

## Abstract

An approach to highly-sensitive mass spectrometry detection of proteins after surface-enhanced concentrating has been elaborated. The approach is based on a combination of mass spectrometry and atomic force microscopy to detect target proteins. (1) Background: For this purpose, a technique for preliminary preparation of molecular relief surfaces formed as a result of a chemical or biospecific concentration of proteins from solution was developed and tested on several types of chip surfaces. (2) Methods: mass spectrometric identification of proteins using trailing detectors: ion trap, time of flight, orbital trap, and triple quadrupole. We used the electrospray type of ionization and matrix-assisted laser desorption/ionization. (3) Results: It is shown that when using locally functionalized atomically smooth surfaces, the sensitivity of the mass spectrometric method increases by two orders of magnitude as compared with measurements in solution. Conclusions: It has been demonstrated that the effective concentration of target proteins on specially prepared surfaces increases the concentration sensitivity of mass spectrometric detectors—time-of-flight, ion trap, triple quadrupole, and orbital ion trap in the concentration range from up to 10^−15^ M.

## 1. Introduction

In modern biomedical researchers, the leading role is played by nanotechnological approaches that enable the detection of biological macromolecules in the range of ultra-low concentrations of 10^−15^ M and lower [1,2]. From a practical point of view, the development trend of analytical approaches with ultra-low concentration sensitivity is forced by the need to identify biomarkers at the early asymptomatic stages of the development of pathological processes when changes in the molecular tracery of the body are insignificant, but drug therapy is most effective [3,4,5].

The use of nanotechnology for early diagnostic tasks is forwarded by the possibility of transferring the biological molecule from solution (volume) to nanochip plane through the interaction of macromolecules (proteins) with a smooth sensory surface [6,7]. The purpose of sensor chips is to enrich and concentrate target protein molecules from the volume of analyzed solution for the subsequent measurement of their physicochemical properties: mass-charged (atomic force microscope), electrochemical (potentiometry, amperometry), and optical [8,9].

Single-molecule detectors such as AFM (atomic force microscopy) and nanowire biosensors use chips with as flat a surface as possible to diminish the effect of surface defects on measurement results. Such sensors make it possible to count and visualize individual proteins and their complexes located on a constrained margin of the chip surface fished from a solution volume. The molecular layer on such chips can be formed by chemical functionalization of the sensor surface or by immobilization of molecular probes, likewise monoclonal antibodies or aptamers [8,10]. So far, the identification of particles concentrated on the surface of the chip is the most severe objection while using the molecular detectors for analytes of biological origin.

Chips for analytical systems based on an atomic force microscope are manufactured from graphite and mica in most, whereas nanometer-sized conductors are made of silicon-on-insulator. The interaction of protein molecules with a small sensor surface enables concentrating the desired analyte from solution in a narrow area for the subsequent detection using single molecular detectors, thus, eliminating the restriction on the lower sensitivity threshold [11]. However, nanotechnology-based quantitative detection of high analyte concentrations in solution is limited by the sensor elements’ physical size and capacity for the analyzed molecules.

This paper summarizes the results of a mass spectrometric analysis of more than 500 samples trapped on the functionalized surface of the chips. The observed opportunities, advantages, and limitations of mass spectrometric study of proteins on the surfaces of nanosensors made it possible to formulate the criteria for selection of targets for highly-sensitive detection. The results were accrued from the proteomic large-scale studies in normal physiological conditions and a wide range of pathophysiological processes, including malignant neoplasms and the cardiovascular and nervous system disorders.

The design of this study included the following stages. First, we performed a mathematical calculation of the possible concentration effect (Section 2.1. Model of Protein Concentration on the Surfaces of AFM Chips). The expected effect of concentration was examined empirically by the matrix-assisted laser desorption and ionization-time of flight-mass spectrometry MALDI-TOF-MS (matrix assisted laser desorption/ionization time-of-flight mass spectrometry) method for the pattern of eight selected globular proteins with different physical and chemical properties and ranged from 10^−9^ to 10^−6^ M (Section 2.2. Experimental Verification of the Concentration-Effect for Several Types of Proteins on the AFM Chips). The success of the empirical examination was verified using various types of mass spectrometric detectors to analyze proteins in the concentration range of 10^−6^–10^−15^ M caught on the surface of AFM chips (Section 2.3. Mass Spectrometric Analysis after Incubation of the Functionalized AFM Chips in Low-Concentration Protein Solutions). Finally, we summarized the advantages and disadvantages of various mass spectrometric detectors (ion trap, triple quadrupole, orbitrap, and time-of-flight detector) for analyzing proteins immobilized on the surface of AFM chips.

## 2. Results

### 2.1. Model of Protein Concentration on the Surfaces of AFM Chips

The work implements the “volume–surface–volume” (3D–2D–3D’) model, which consists of enriching the target analyte molecules in a small volume for the subsequent identification. In this model, the target analyte of a wide range concentration is concentrated from solutions on a small functionalized surface of the chip. The analyte molecules are then transferred into a simple solution of a small volume in an amount sufficient for mass spectrometric identification.

Atomically smooth chips with functionally active chemical groups (mica with –NH_2_ groups) were used as substrates in the experimental design. The studied protein molecules were concentrated and covalently bound on the surface of substrates, i.e., the so-called “chemical fishing” procedure [12].

**Figure 1:**
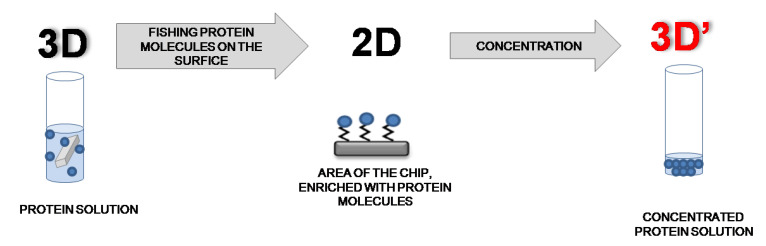
The concentration model “volume–surface–volume” (3D–2D–3D’). The tube contains an analyte solution and a chip with a functionalized surface (3D). The surface of the chip is enriched with protein molecules due to the formation of covalent bonds (“chemical fishing”) (2D). Elution of protein molecules (fragments) from the surface of the chip into a small volume of solution (3D’) for mass spectrometric measurements.

The usage of functionalized surfaces (2D) permits one to efficiently concentrate protein molecules on a small area of a chip from a solution (3D) in an amount sufficient for the subsequent mass spectrometric analysis (3D’) [4,5,7] (Figure 1).

In the case of chemical fishing, molecules of analyte were concentrated through the covalent interaction with the on-surface functional groups. For this purpose, a flat chip with a functionalized surface (working area) was incubated in a solution with an analyte. Protein molecules were concentrated on a small working area whereupon bounded molecules of analyte (proteins) were treated with trypsin for digestion, and the resulting fragments (peptides) were eluted into a small volume (3D’). In the proposed model, under the assumption that the molecules are organized as a compact monolayer, the concentration factor (*F*) of the target analyte can be calculated as following [1]:

$$F=\;\frac{C^{\prime }}{C_{0}}\;=\;\frac{n^{\prime }/V^{\prime }}{n_{0}/V_{0}}\;=\frac{N_{scan}}{V^{\prime }}\times \frac{V_{0}}{N_{0}}$$

where *C*_0_*, n*_0_—protein concentration and amount of substance in the initial solution (3D); *C′, n′*—the eluted protein concentration and amount of substance in the solution (3D’); *N*_0_, *V*_0_—the number of protein molecules and the volume of the initial solution; *N_scan_, V′—*the number of proteins on the chip surface detected by AFM scanning and the volume of the final solution, respectively.

Following the above scheme, we express the number of molecules (*N_scan_*) in the final solution that can be visualized in assistance with AFM calculation relative to the maximum possible number of particles (*N_sat_*), which is determined by the capacity of the functionalized area of the chip (Equation (1)):

(1) $$\{\begin{array}{c} N_{0}\;\leq \;N_{sat},\;if\;N_{scan}\;=\;N_{0}\\ N_{0}\;>\;N_{sat},\;if\;N_{scan}\;=\;N_{sat} \end{array}$$

where *N_sat_* is the number of protein molecules organized as a monolayer on the surface of the functionalized area of the chip. To follow from the surface to volumetric quantities, we expressed the number of particles (molecules) in the initial solution (*N*_0_) and the final solution (*N_scan_*) through the molar concentrations *C*_0_ and *C′*, respectively:

*N*_0_ = *C*_0_ × *N_a_*× *V*_0_, where *N_a_* is the Avogadro number (6.02 × 10^23^ molecules), (2a)

*N*_*scan*_ = *C′* × *N*_*a*_ × *V′* (2b)

The capacity of the functionalized surface of the chip corresponds to (*N_sat_*):

(3) $$N_{sat}\;=\;C_{sat}\;\times \;N_{a}\times \;{V_{0}}_{\;}\Rightarrow \;C_{sat}=\;\frac{N_{sat}}{N_{a}\times V_{0}}$$

Similarly to the equations system (1) for the number of particles, we determined volumetric characteristics for the initial solution conditions with a concentration of molecules below and above the saturating concentration (*C_max_*).

(4) $$\{\begin{array}{c} C_{0}\;\times \;N_{a}\;\times \;V_{0}\;<\;C_{max}\;\times \;N_{a}\;\times \;V_{0}\;\Rightarrow \;C^{\prime }\;\times \;N_{a}\;\times \;V^{\prime }=\;C_{0}\;\times \;N_{a}\;\times \;V_{0}\\ C_{0}\;\times \;N_{a}\;\times \;V_{0}\;>\;C_{max}\;\times \;N_{a}\;\times \;V_{0}\;\Rightarrow \;C^{\prime }\;\times \;N_{a}\;\times \;V^{\prime }=\;C_{max}\;\times \;N_{a}\;\times \;V_{0} \end{array}$$

(5) $$\begin{array}{c} \{\begin{array}{c} C_{0}\;\times \;V_{0}\;<\;C_{max}\;\times \;V_{0}\;\Rightarrow \;C^{\prime }\;\times V^{\prime }=\;C_{0}\;\times V_{0}\\ C_{0}\;\times \;V_{0}\;>\;C_{max}\;\times \;V_{0}\;\Rightarrow \;C^{\prime }\;\times V^{\prime }=\;C_{max}\;\times V_{0} \end{array}\Rightarrow \\ \{\begin{array}{c} C_{0}\;<\;C_{max}\;\Rightarrow \;\frac{C^{\prime }}{C_{0}}=\;\frac{V_{0}}{V^{\prime }}\;\Rightarrow \;C^{\prime }=\;C_{0}\;\times \;\frac{V_{0}}{V^{\prime }}\\ C_{0}\;>\;C_{max}\;\Rightarrow \;\frac{C^{\prime }}{C_{max}}=\;\frac{V_{0}}{V^{\prime }}\;\Rightarrow \;C^{\prime }=\;\frac{V_{0}}{V^{\prime }}\;\times \;C_{max} \end{array} \end{array}$$

**Figure 2:**
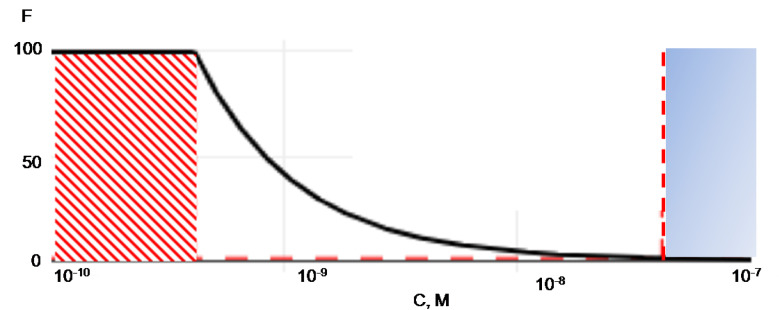
The dependence of the concentration factor (F) and protein concentration in the stock solution (in volume). Conditions for calculating the concentration factor dependencies are normalized to 1 mm^2^ functionalized surface area of the chip, 1 mL volume of the initial solution (3D) with the analyte, and 10 μL volume of the final solution (3D’) with the analyte.

After transformation of Equations (4) and (5), the value of the concentration factor (*F*) can be expressed as a system of equations (as seen in Equation (6) of a linear dependence for the initial concentration below the saturating concentration (*C_max_*), and an exponentially decaying dependence for *C*_0_ > *C_max_* (Figure 2).

(6) $$F=\{\begin{array}{c} \frac{V_{0}}{V^{\prime }},\;if\;C_{0}\;<\;C_{max}\;\\ \frac{V_{0}}{V^{\prime }}\times \frac{C_{max}}{C_{0}},\;if\;C_{0}\;>C_{max} \end{array}$$

The equations system (as seen in Equation (6) can be illustrated as a dependence of the concentration factor (*F*) on the ratio of the initial and final volumes of the solution with the analyte or the initial concentration of the analyte in solution (Figure 2).

As demonstrated in Figure 2, with increasing analyte concentration in a stock solution, the F value declines. The dependence of F on the ratio of initial to final volumes (*V*_0_/*V′*) is linear, and the slope is determined by the ratio between the saturating concentration (*C_max_*) and the concentration of the analyte in the initial (*C*_0_) solution. Whether the analyte concentration is below the *C_max_*, the slope of the curve in logarithmic scales is not affected by the concentration and is equal to 45° (Figure 2). With the increase of the concentration of the analyte, the slope of the straight-line decreases. The concentration factor does not depend on the analyte concentration in the solution if represented below the *C_max_*. As the concentration of analyte increases, the value of *F* decreases exponentially. At a 10^−7^ M concentration, the effect is leveled since *F* assumes values of less than 1. Conditions illustrating *F* behavior in Figure 2 suggest that the concentration effect is not observed for analyte solutions with concentrations above 10^−7^ M, which correspond to a high-copy protein range. In this range of concentrations, it is advisable to use developed surfaces (microbeads, chromatographic columns) with a high capacity (blue zone in Figure 2). On the contrary, if the initial concentration is below the *C_max_* value, the concentration factor (F) function’s saturation is expectedly observed (red region, Figure 2).

### 2.2. Experimental Verification of the Concentration-Effect for Several Types of Proteins on the AFM Chips

To verify the concentration factor calculated values, we performed experiments using eight types of globular proteins with different physical and chemical properties (see Materials and Methods, Table 1).

**Figure 3:**
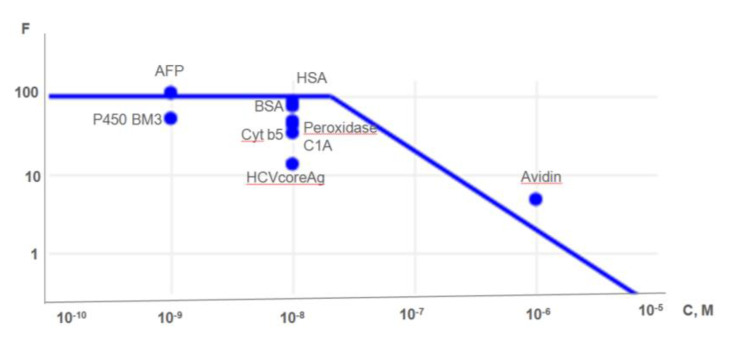
Dependence of *F* on the concentration (in logarithmic scales) of protein in solution. Experimental conditions: the functionalized surface area of the chip is 1 mm^2^, the volume of the initial solution (3D) of the analyte is 1 mL, the volume of the final solution (3D’) of the analyte is 10 μL. The protein concentration on the chip surface was conducted as a chemical fishing, and measurements of the signal were performed on an Autoflex III mass spectrometer (time of flight—TOF). The description of proteins is presented in Table 1.

The theoretically predicted curve characterizing the concentration function (*F*) in logarithmic scales and estimated according to the equations systems ((as seen in Equation (6)), is shown in Figure 3 (Section 2.1. Model of Protein Concentration on the Surfaces of AFM Chips). Points located near the theoretical curve indicate the empirically determined lowest globular protein concentrations with various origins (humans, bovine, plant, viruses C).

We analyzed solutions with analytes in a range from 10^−10^ to 10^−5^ M, and the lowest concentrations detected by the MALDI-TOF-MS approach are demonstrated in Figure 3. The obtained experimental results fall within the calculated theoretical one and endorse the concentration-effect undergone on the functionalized AFM chip surfaces. The function break at 5 × 10^−7^ M is caused by the saturation of the functionalized surface at a certain condition (Figure 3). It has been determined that the higher concentration of the analyzed protein, the lower concentrating effect (F) was observed with almost complete diminishing from the 10^−5^ M and higher in accordance with the Equation (6). The regression curve (Figure 3) is represented by the combination of two linear curves that feature the concentrating effect. In one case, when the original concentration of analyte is below the saturation concentration, the equation ($C_{0}\;<\;C_{max})$ is satisfied, F = 100, and the function fits *f = k × x.* In another case, when the original concentration of analyte exceeds the saturation concentration ($\;C_{0}\;>C_{max}$) it led F to the zero-point and the dependence function fits *f = k × x + b*.

### 2.3. Mass Spectrometric Analysis after Incubation of the Functionalized AFM Chips in Low-Concentration Protein Solutions

To examine the conditions of close to matter-of-the-fact for preliminary preparation of a sample, the modes of mass spectrometric measurements and the interpretation of the results obtained, we designed the analysis of proteins composition that were trapped on the surface of mica chips after incubation in solutions fortified with analytes in a range of 10^−5^–10^−15^ M. The AFM chip contained two zones—a chemically functionalized sensory and unmodified control zone.

The mass spectrometric analysis was carried out for four types of proteins being distinct in their origin, molecular weight, number of cleavage sites and their spatial accessibility, and degree of the amino acid sequence hydrophobicity (ratio of hydrophobic amino acids to hydrophilic). The examined proteins were covalently immobilized on the AFM chip’s functionalized surface (Table 2). The visualized molecules were counted using AFM software (Pleshakova et al., 2017). The number of objects recorded by AFM on the mica chips’ surface for all on-surface trapped proteins was on average 3 × 10^8^ ± 1 × 10^8^. In contrast, the minimum recorded concentration of proteins in the incubation solution was 10^−15^ M.

It should be noted that if protein molecules are immobilized on a small area of surfaces, the sensitivity of detection is increased by at least one order of magnitude compared to measurements of the corresponding analyte in solution. Thus, the number of particles recorded by AFM on the functionalized surface is sufficient for the successful mass spectrometric measurements.

**Figure 4:**
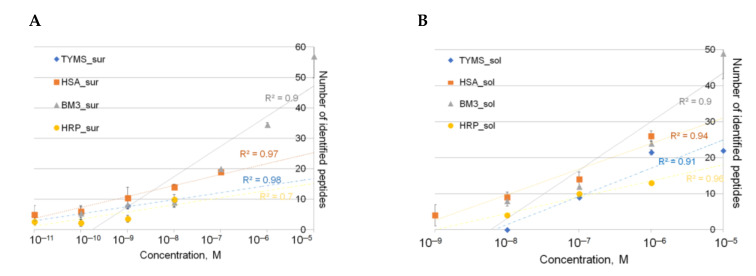
Dependence of the number of detected protein peptides on the protein concentration in solution (**A**) and proteins immobilized on the surface with a molecular relief (**B**). Measurements were taken using matrix-assisted laser desorption and ionization-mass spectrometry (MALDI-TOF-MS) on an Autoflex III instrument (TOF). Measurement conditions: 2000 laser shocks, laser intensity, and frequency 90% and 60 Hz, respectively.

Calibration dependencies between the number of identified peptides and the mass spectrometric signal (TOF) of the target protein were determined for conditions of surfaces with molecular relief (mica) and of the desired protein content in the solution of the analyte. The dependence was plotted for thymidylate synthase (P04818), human serum albumin (P02768), cytochrome P450 BM3 (P14779), and horseradish peroxidase (P00433) covalently immobilized on the chemically activated surface (S = 0.5 cm^2^) (Figure 4).

Based on the obtained results (Figure 4), the higher the protein concentration in the solution, the greater the number of peptides that can be registered. Concentration sensitivity (TOF) in solution is determined at a level of up to 10^−8^ M and up to 10^−11^ M if measurements performed after incubation of the functionalized surface in the analyte solution. Using an ion trap-type mass spectrometer (IT), it is possible to successfully identify the designed proteins in a solution with a concentration of up to 10^−9^ M and up to 10^−10^ M if washed from the surface. If measurements are performed on an orbital trap-type LTQ XL mass spectrometer (OT), the sensitivity is about 10^−6^ M for both the solution and surface washings.

**Figure 5:**
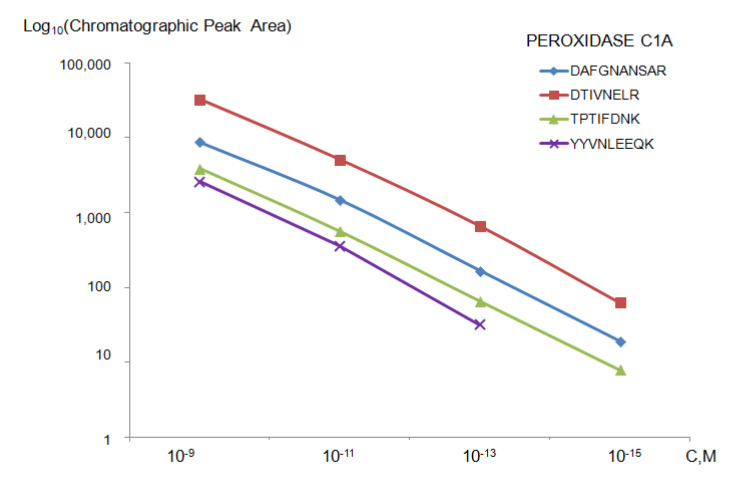
The dependence of the chromatographic peak areas plotted for four peptides on the concentration of horseradish peroxidase (Peroxidase C1A) in the incubation solution in a range of 10^−9^–10^−15^ M. Measurements were performed on a QqQ, Agilent 6495 LC/MS triple quadrupole mass spectrometer.

**Figure 6:**
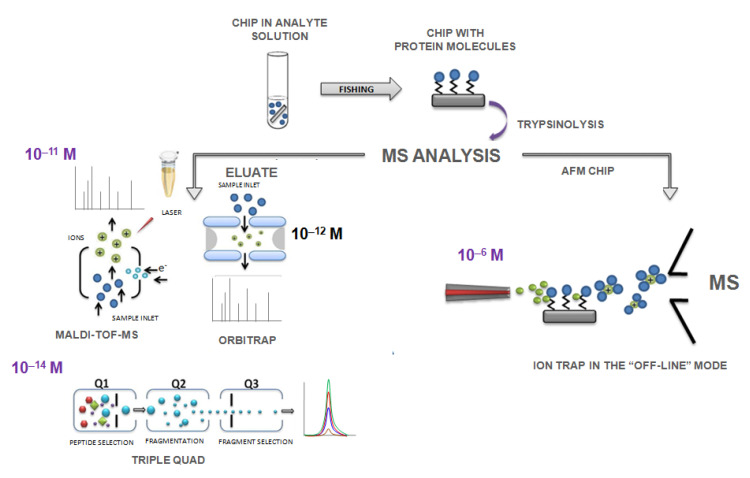
Mass spectrometric (MS) analysis of protein molecules concentrated on the surface with molecular relief. Two methods of MS analysis: (1) elution of tryptic peptides from the surface of the molecular-chip (TOF, IT, OT, QqQ) and (2) direct MS analysis from the surface of the chip (IT).

If measurements are taken on a triple quadrupole Agilent 6495 Triple Quadrupole LC/MS mass spectrometer (QqQ), it is possible to identify proteins with a concentration of up to 10^−13^ M in the solution and up to 10^−15^ M for washings from the surface (Figure 5). Evidently, the higher the protein concentration in the solution of analyte (10^−9^ M), the larger the chromatographic peak area for target components (Figure 6).

Thus, the best concentration sensitivity of 10^−15^ M was achieved using the targeted SRM/MRM (selected reaction monitoring/multiple reactions monitoring) approach on a triple quadrupole mass spectrometer for horseradish peroxidase (HRP) protein if peptide fragments were detected from the surface of the AFM chip.

## 3. Discussion

### Possibility of Using Various Types of Detectors for Analyzing Proteins from the AFM Chips

Mass spectrometric detection of protein composition on a chip’s surface with a molecular relief provides for at least two experimental approaches (Figure 6). The first method supplies preliminary eluting of the hydrolyzed sample from the chip’s surface for subsequent mass spectrometric measurements (TOF, IT, OT, and QqQ). The second method performs the direct mass spectrometric measurements of the analytes mixture directly from the chip’s surface (IT) (Figure 6).

Table 1 compares the experimental characteristics for protein detection (Table 2) among mass spectrometric systems used in biomedical research using two different method of sample preparations (Table 3). Selected analytical techniques are different in types of ionization and mass analyzer (see Table 4 and Table 5). The result of mass spectrometric measurements for the analyzed sample is a scan (TOF and IT) or a scan, and a chromatographic peak (IT, OT, and QqQ) comprises the information about the intensity and mass-to-charged characteristics of peptide ions and their fragments or transitions (peptide ion fragmentation spectrum). The preparation of a surface with a molecular relief is carried out according to two methods before mass spectrometric analysis. According to the first approach, MS measurements were performed for the dissolved analyte (eluate) by dropping it onto a MALDI target (TOF) or loading it into a chromatographic system for separation and concentration (IT and QqQ). The second approach was an off-line mass spectrometric analysis of the protein composition directly from the functionalized surface with a molecular relief (chip) (IT). According to this technique, the electrospray cloud contacts the chip’s surface and traps analyte ions, and takes them into the mass analyzer (see Figure 6 and Table 2).

It was found (Table 1) that combination of a mass spectrometric detector with a chromatographic system is preferable in sensitivity for protein analysis of the eluate from the surface of the chip compared to TOF, as well as nano-ESI IT (electrospray ionization ion trap mass spectrometry), by at least 2–3 orders of magnitude (Table 1, line 5).

Even though in IT with off-line nano-ESI, analyte molecules directly enter the MS analyzer from the chip surface and bypass elution and dilution stages, the sensitivity of this method is significantly lower compared to MS systems coupled to HPLC systems. The low flow rate explains the observed effect in the IT analytical system (only 0.001 μL/min or 0.017 nL/s). The analyzed sample does not concentrate on the chromatographic column but smoothly spreads and enters the mass spectrometric analyzer at an extremely low speed. Each MS scan contains the target peptide in an amount of only 0.003% of the input. In contrast, in the HPLC-MS/MS systems, the target peptide is intensely concentrated on a chromatographic column, and the target compound is eluted in a narrow time window for about 20 s.

Moreover, each consequent MS scan increments the peptide content exponentially along with the characteristic chromatographic tailing from the peak base to apex. In the case of IT, there is an option to interpret MS scans close to the peak apex, which is “enriched” with the target peptide. The QqQ offers the option of integrating the accumulated scans within the characteristic chromatographic peak width.

It is assumed that the HPLC-MS systems provide almost complete elution of the target peptide from the chromatographic column constrained within a single peak. In the case of IT and QqQ, one scan (assuming the compounds are eluted uniformly from the column) embodies at least about 4% of the target peptide of the inlet. Thus, we showed that the coupled HPLC-MS systems are of three orders of magnitude more sensitive than the off-line IT (direct sample entry into the MS). Besides, the HPLC system and QqQ are preferable to the rest of the systems due to permitting to account the resulting chromatographic peak as an integral item with accumulated scans. An obvious drawback of HPLC-MS systems compared to direct sample injection is that the long separating gradient lasted from 20 to 90 min.

**Figure 7:**
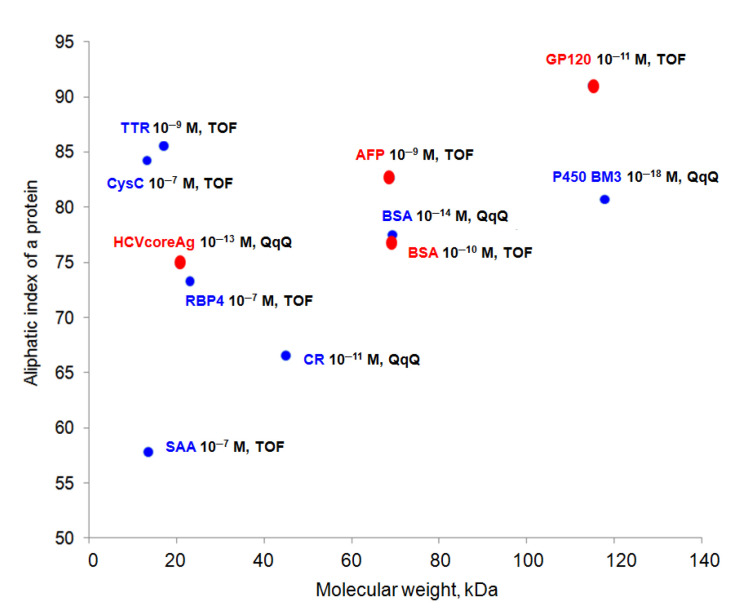
The sensitivity of mass spectrometric methods for the detection of proteins concentrated on the surface of mica from solutions of the analyte with initial concentrations of less than 10^−5^ M (red dots) and concentrated on the other types of surface or sorbents from solutions of the analyte with initial concentrations of less than 10^−5^ M (literature data, blue dots). The dot volume displays the minimal recorded protein concentration, so the larger the dot size, the higher the concentration.

Indeed, mass spectrometric results confirm the effect of protein concentration using the immobilization procedure on the AFM chip, which complies with the previously published data acquired from other types of affinity carriers (microbeads) (Figure 7).

As demonstrated (Figure 7, red dots), the best concentration sensitivity at the level for proteins concentrated on the surface of the AFM chip using affinity methods was achieved for objects characterized by different molecular weights from 20 to 120 kDa and an aliphatic index value from 75 to 91—glycoprotein HIV-1 gp120 (LOD 10^−11^ M and TOF), human serum albumin (HSA, LOD 10^−9^ M, IT, and TOF), bovine serum albumin (BSA, LOD 10^−10^ M, and TOF), core antigen of viral hepatitis C (HCVcoreAg, LOD 10^−13^ M, and TOF), horseradish peroxidase (HRP, LOD 10^−15^ M, and QqQ). In the literature (blue dots in the figure), using immunoaffinity fishing and monoclonal/polyclonal antibodies (magnetic microsized spheres, nozzles for automatic pipetting), most of the blood plasma proteins were recorded at concentrations of 10^−7^–10^−9^ M for TOF and 10^−11^–10^−18^ M for QqQ, including transthyretin (TTR, LOD 10^−9^ M, and TOF), cytochrome P450 BM3 (P450 BM3, LOD 10^−18^ M, and QqQ) [12], bovine serum albumin (BSA, LOD 10^−14^ M, and QqQ) [12], serum amyloid A (SAA, LOD 10^−7^ M, and TOF) [13], cystatin C (CysC, LOD 10^−9^ M, and TOF) [14], calreticulin (CR, LOD 10^−11^ M, and QqQ) [15]. The physicochemical properties of proteins presented in the literature are also different. Thus, the molecular weight of the described proteins ranges from 17 to 120 kDa and the aliphatic index values are from 58 to 85.

## 4. Materials and Methods

The mica (SPI, West Chester, PA, USA) surfaces were used in this study for the concentration of molecular targets (Table 3)

Dry trypsinolysates were dissolved in 10 μL of a 0.7% TFA solution to perform mass spectrometric measurements (MALDI-MS) on an Autoflex III (Bruker, Bremen, Germany) and dissolved in 10 μL of a 0.1% formic acid solution to make tandem measurements with the electrospray type of ionization.

Chromatographic systems comprised of the following elements: Agilent 1200 (Agilent, Paolo Alto, CA, USA), Chip Cube (Agilent, Paolo Alto, CA, USA), Ultimate 3000 Nano-flow (Thermo Scientific, Waltham, MA, USA).

The calibration of mass spectrometers with electrospray type of ionization was carried out following the manufacturer’s recommendations. TOF was calibrated using a peptide calibration standard (Peptide Calibration Standard, Bruker Daltonics, Bremen, Germany).

Tandem mass spectra were analyzed using proteomic search engines: OMSSA (Geer et al., 2004), Mascot, X!Tandem (Beavis et al., 2004). MALDI mass spectra were identified using Mascot MRP. The search was performed for inverted and random amino acid sequences (decoy). Identification settings for the selected MRPs were similar: database—“Swiss-Prot”; enzyme trypsin; the charge state of peptide ions is 2+, 3+, and 4+; the number of missed hydrolysis sites is not more than 1; the accuracy of measurements of peptide ions is not more than 200 ppm for LC/MSD Trap XCT Ultra and LTQ XL, 10.0 ppm for Q Exactive; the accuracy of measurements of fragment ions is not more than 0.05 Da; fixed modification—cysteine, pyridylethylation, oxidized methionine as a variable modification; the number of detected peptides is not less than 2. Peptide spectra matches percentage of false-positive results (false discovery rate, FDR) not more than 1%. Mass spectrometric measurements and bioinformatics work were performed using the equipment and computer cluster of the “Human Proteome” Core Facility (IBMC, Moscow, Russia).

Reagents: acetonitrile, isopropanol, formic acid (ACROS, Morris Plains, NJ, USA), trifluoroacetic acid (TFA), ammonium bicarbonate, urea (Sigma, Raleigh, NC, USA), α-cyano-4-hydroxycinnamic acid (HCCA) (Bruker Daltonics, Bremen, Germany), trypsin (Promega, Madison, WI, USA), methanol, deionized water purified using Millipore Simplicity UV (France), and ethanol (Reachim, Moscow, Russia).

## 5. Conclusions

The use of smooth surfaces (chips) seems to be relevant for highly sensitive protein detection. Such chips make it possible to control the surface quality when performing functionalization procedures, including using molecular probes (aptamers or partner proteins), when forming molecular objects when executing chemical or biospecific immobilization procedures, washing efficiency, and to monitor the state of aggregation of the studied object. Limits of the sensory surface’s geometric dimensions require the development of methods for the adequate enrichment of protein molecules from the volume to an amount sufficient for subsequent detection and identification. In response to this challenge, molecular detectors appeared—atomic force microscope and nanowire biosensors, which allow you to visualize, count, and detect protein molecules in real-time in solutions with low (<10^−^^9^ M) and ultra-low (≤10^−14^ M ) concentrations. In practice, the restriction of the use of molecular detectors is such that detectors cannot identify proteins and their complexes, which is especially important in studies of complex protein mixtures, including biological origin. The development of mass spectrometric protein analysis, complementing the capabilities of nanotechnological devices, opens up fundamentally new opportunities for biomedical research.

In the present study, the possible effect of concentrating the target proteins on the surfaces was calculated and experimentally confirmed for a wide range of proteins with different physical and chemical properties. It has been shown using the most regnant mass spectrometric detectors in the biomedical field that the procedure for the initial concentration of proteins on the surface makes it possible to increase the sensitivity of the protein detection by about 1–2 orders in comparison with that of analyses without using surfaces.

## Figures and Tables

**Table 1:** Target proteins used in the study.

Section Number	Protein (UniProt AC)	Mw (kDa)	Taxon	Manufacturing
2.2	Alpha-fetoprotein (P02771)	68.7	Human	USBio (USA)
2.2	Serum albumin (P02768)	69.4	Human	Agilent (USA)
2.2, 2.3	Peroxidase C1A (P00433)	38.8	Armoracia rusticana	Sigma (USA)
2.2, 2.3	Serum albumin (P02769)	69.3	Bovine	Sigma (USA)
2.2	Cytochrome b5 (P00167)	14.3	Human	provided by prof. S.A. Usanov, Institute of Bioorganic Chemistry (Republic of Belarus)
2.2, 2.3	Bifunctional cytochrome P450/NADPH-P450 reductase (P14779)	117.8	*Bacillus megaterium*	provided by prof. A.V. Munro, University of Manchester (UK)
2.3	Thymidylate synthase (P04818)	35.7	Bull	USBio (USA)
2.2	Avidin (P02701)	16.8	Human	Agilent (USA)

**Table 2:** Comparison of types of mass spectrometric systems utilized for protein detection.

№	Characteristic	Autoflex III (TOF)	LC/MSD Trap XCT Ultra (IT)	Agilent 6495 Triple Quadrupole LC/MS (QqQ)	LTQ XL (nanoESI, IT)
1	Sample volume, μL	1	1	1	1
2	Peak width at the base, sec	N/A	25	24	–
3	MS/MS scan, sec	N/A	12	0.8	1.7
4	Flow rate, μL/min (μL/s)	N/A	0.2 (0.003)	3 (0.05)	0.001 (1.7 × 10^−5^)
5	The average amount of protein in one MS/MS scan per chromatographic peak, %	<0.001	3.6	~0.4	0.003
6	Total analysis time, min	<1	30	80	<2

N/A” (not availible).

**Table 3:** Methods of chip preparation for mass spectrometric measurements.

Chip	Configuration	Tripsinolysis	Ref.
Mica	1 work/control zone	Volume 8 μL:150 mM NH_4_HCO_3_, Acetonitrile 1%, 0.5 M guanidine hydrochloride, glycerol 10% (pH 7.5–8.0), and 1.5 μL of a solution of modified trypsin with a concentration of 0.1 μM.	[5]
	1–3 work zones/1–2 control zones	70 μL volume:150 mM NH_4_HCO_3_, 1% acetonitrile, 0.5 M guanidine hydrochloride, 10% glycerol (pH 7.5–8.0), and 2 μL of modified trypsin	[7]

**Table 4:** Types of the mass spectrometric detectors.

Name	Mass Spectrometer	Ion Source	Ionization	Detector
TOF	Autoflex III	MALDI * chip	MALDI	Time of Flight
IT	LC **/MSD Trap XCT Ultra	Chip Cube	Electrospray	Ion Trap
OT	Q Exactive	Ultimate 3000 Nano-flow		OrbiTrap
QqQ	Agilent 6495 Triple Quadrupole LC/MS	Agilent 1200		Triple Quad
IT	LTQ XL	nanoDESI (Direct ESI MS)		Ion Trap

MALDI *—matrix-assisted laser desorption and ionization; LC **—liquid chromatography.

**Table 5:** Parameters of mass spectrometric measurements.

Mass Spectrometer	Mobile Phases: Solution A and B	Gradient (min)	Measurement Parameters			
			Ionization	M/Z for MS1; MS2	MS1 Spectrum Accumulation	Software
TOF, Autoflex III	–	–	positive	750–3000; NA	10,000 laser shots	flexAnalysis 2.0 (Bruker Daltonics, Germany)
IT, LC/MSD Trap XCT Ultra	0.1% formic acid in distilled water; 0.1% formic acid in 90% acetonitrile	80	positive	400–1200; 200–1350	100,000	Data Analysis 3.3 (Bruker Daltonics, Germany)
OT, Q Exactive	0.08% formic acid; 0.015% trifluoroacetic acid; 0.08% formic acid, 0.015% trifluoroacetic acid in acetonitrile	80	positive	420–1250; 200–1350	500,000	Mass Hunter B2.0
QqQ, Agilent 6495 Triple Quadrupole LC/MS	0.1% formic acid in deionized water; 0.1% formic acid in a solution of 90% acetonitrile	75	positive	determined by selected transitions	100,000	Mass Hunter Qualitative Analysis B2.0
IT, LTQ XL	Direct nanoDESI	−	positive	420–1250; 100–1350	100,000	Data Analysis 3.3 (Bruker Daltonics, Germany)

−: No gradient.

## Data Availability

Data are available from the corresponding author upon request.

## Abbreviations

AFM	atomic force microscopy
HCCA	α-cyano-4-hydroxycinnamic acid
IT	ion trap
LC	liquid chromatography
LOD	limit of detection
MALDI	matrix-assisted laser desorption and ionization
MS	mass spectrometer, mass spectrometry
OT	Orbitrap
TFA	trifluoroacetic acid
TOF	time of flight
